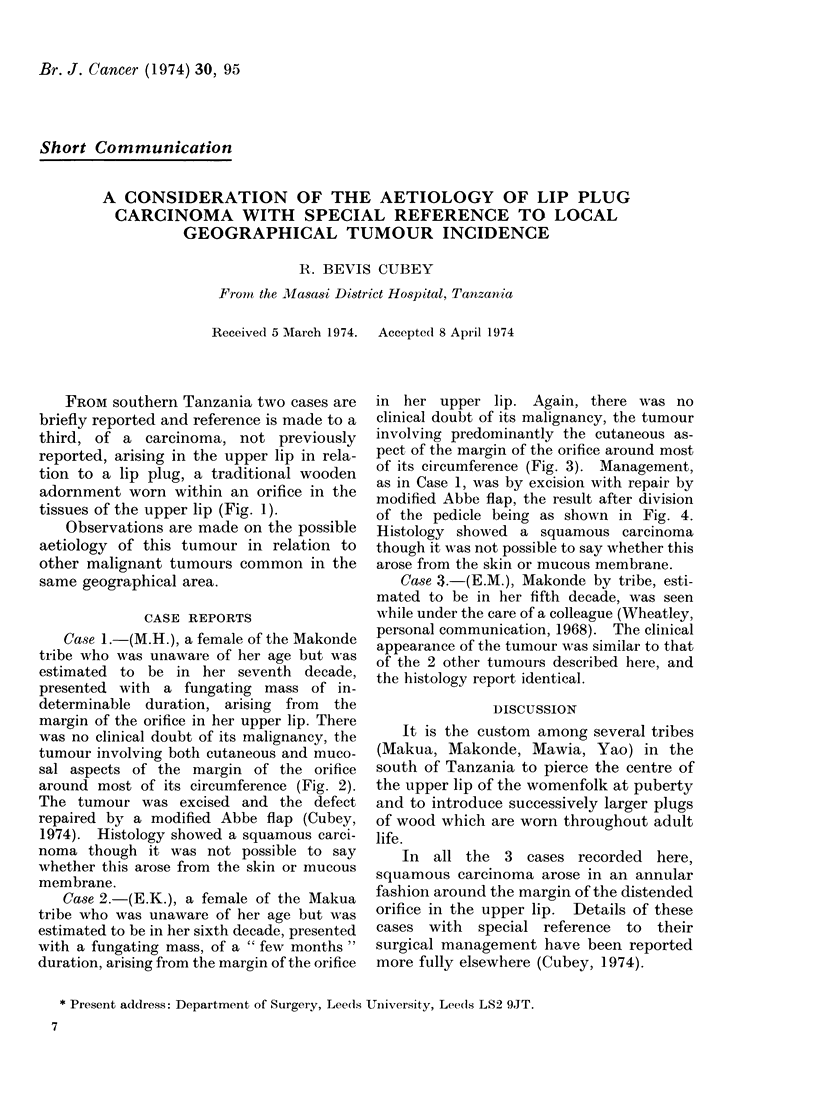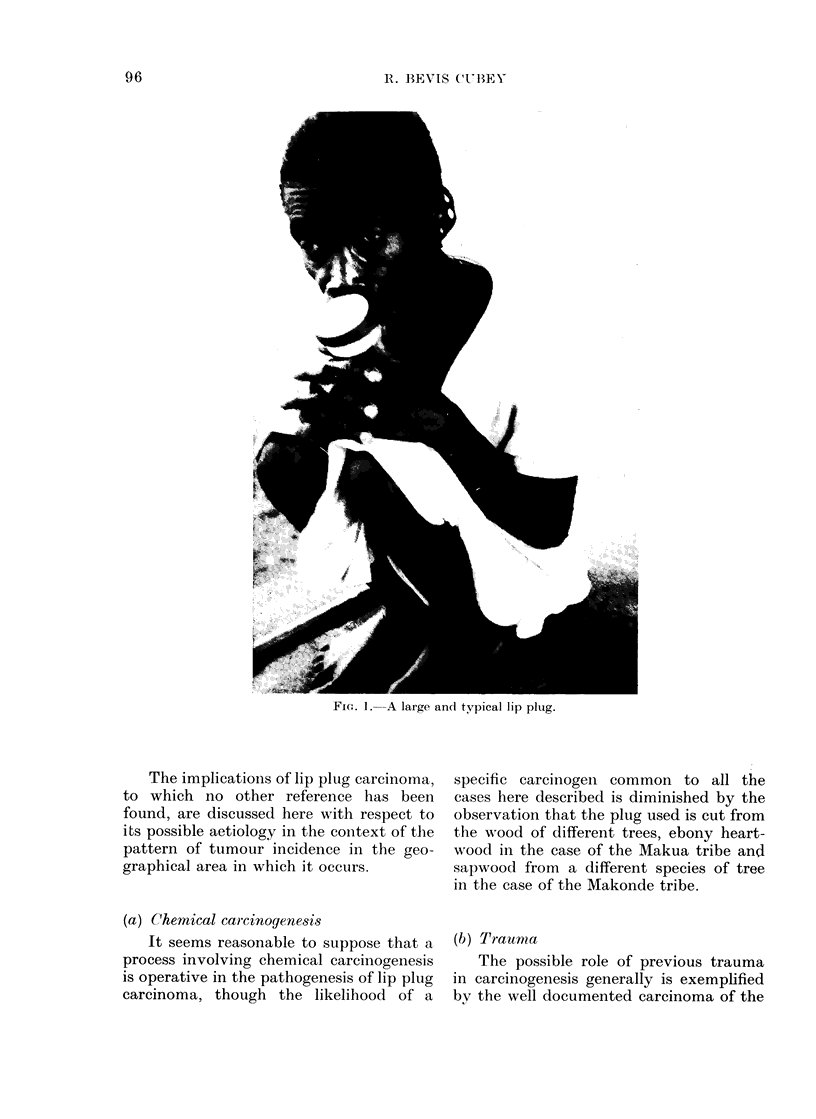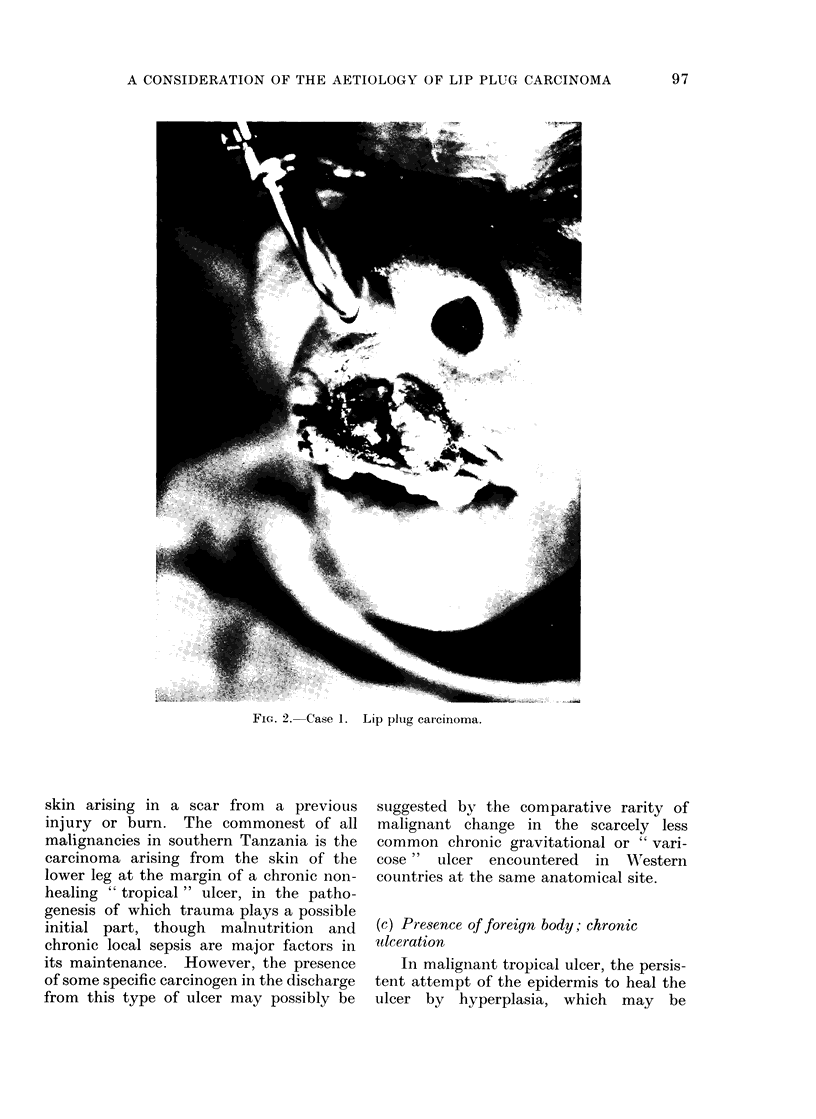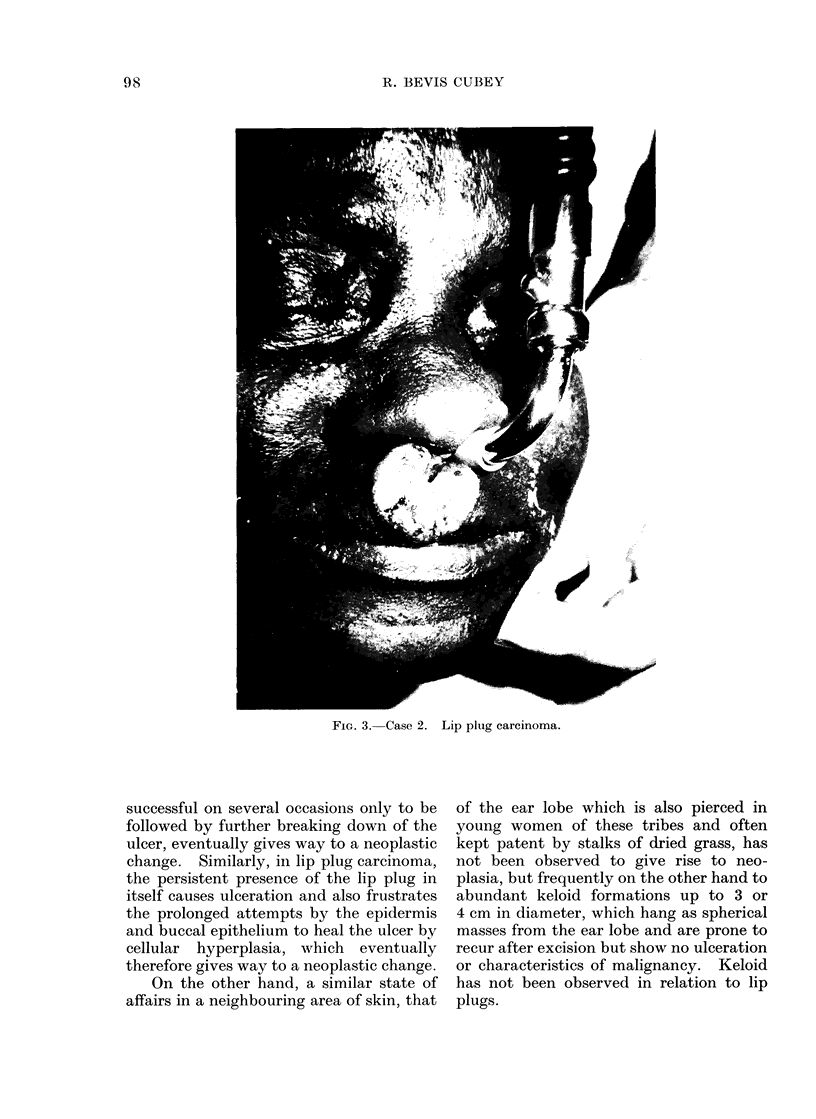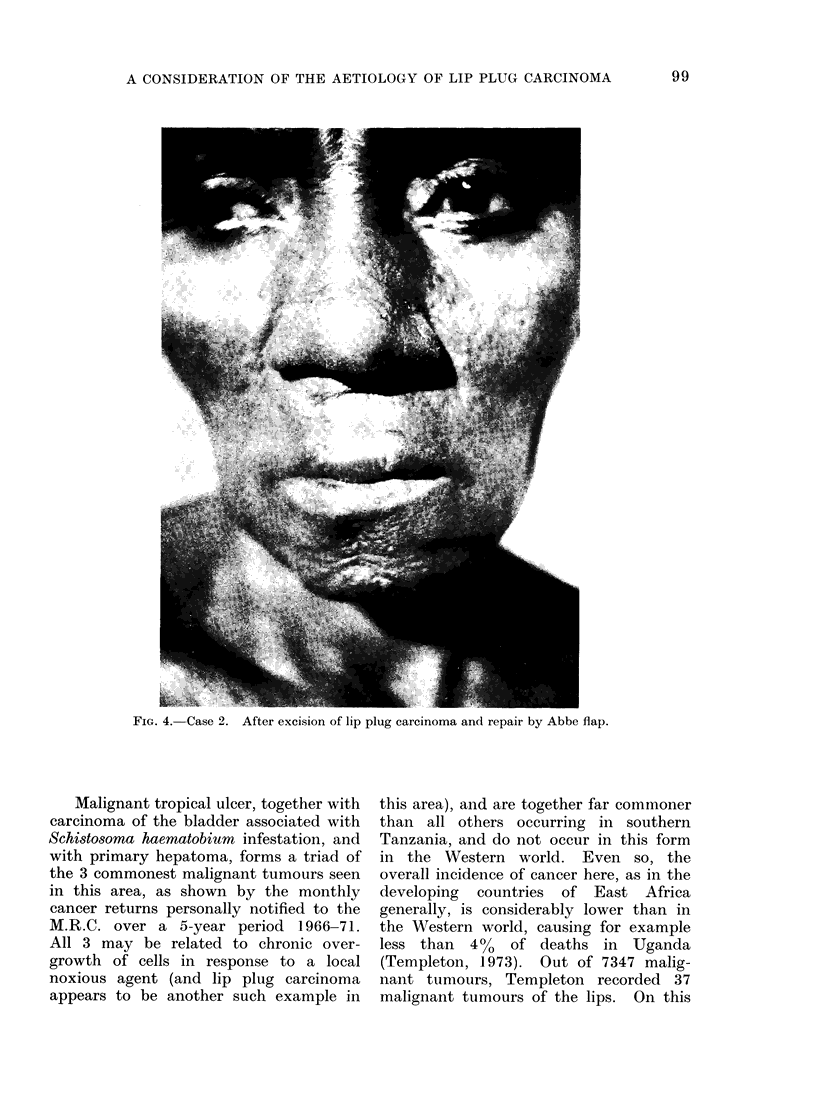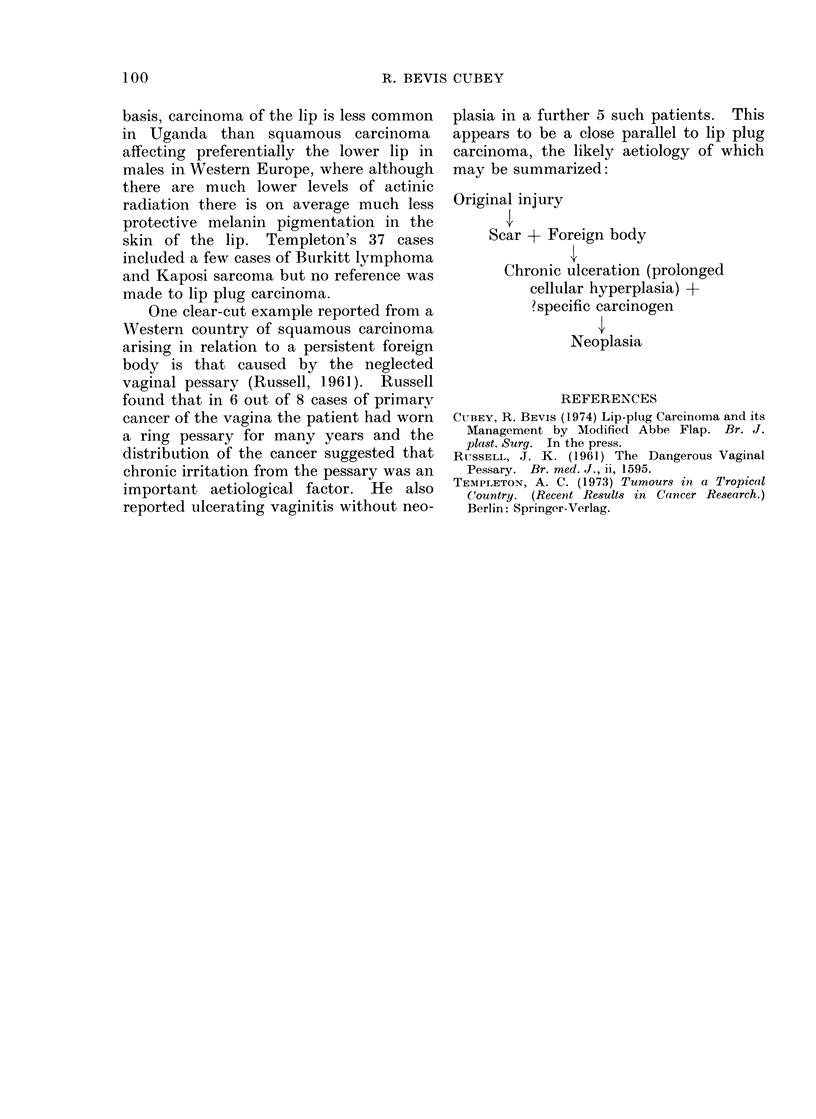# A consideration of the aetiology of lip plug carcinoma with special reference to local geographical tumour incidence.

**DOI:** 10.1038/bjc.1974.117

**Published:** 1974-07

**Authors:** R. B. Cubey

## Abstract

**Images:**


*Br. J. Cancer* (1974) **30**, 95

***Short Communication***

### A CONSIDERATION OF THE AETIOLOGY OF LIP PLUG CARCINOMA WITH SPECIAL REFERENCE TO LOCAL GEOGRAPHICAL TUMOUR INCIDENCE

R. BEVIS CUBEY

*From the Masasi District Hospital, Tanzania*

Received 5 March 1974. Accepted 8 April 1974

FROM southern Tanzania two cases are briefly reported and reference is made to a third, of a carcinoma, not previously reported, arising in the upper lip in relation to a lip plug, a traditional wooden adornment worn within an orifice in the tissues of the upper lip (Fig. 1).

Observations are made on the possible aetiology of this tumour in relation to other malignant tumours common in the same geographical area.

### CASE REPORTS

*Case* 1.—(M.H.), a female of the Makonde tribe who was unaware of her age but was estimated to be in her seventh decade, presented with a fungating mass of indeterminable duration, arising from the margin of the orifice in her upper lip. There was no clinical doubt of its malignancy, the tumour involving both cutaneous and mucosal aspects of the margin of the orifice around most of its circumference (Fig. 2). The tumour was excised and the defect repaired by a modified Abbe flap (Cubey, 1974). Histology showed a squamous carcinoma though it was not possible to say whether this arose from the skin or mucous membrane.

*Case* 2.—(E.K.), a female of the Makua tribe who was unaware of her age but was estimated to be in her sixth decade, presented with a fungating mass, of a " few months " duration, arising from the margin of the orifice in her upper lip. Again, there was no clinical doubt of its malignancy, the tumour involving predominantly the cutaneous aspect of the margin of the orifice around most of its circumference (Fig. 3). Management, as in Case 1, was by excision with repair by modified Abbe flap, the result after division of the pedicle being as shown in Fig. 4. Histology showed a squamous carcinoma though it was not possible to say whether this arose from the skin or mucous membrane.

*Case* 3.—(E.M.), Makonde by tribe, estimated to be in her fifth decade, was seen while under the care of a colleague (Wheatley, personal communication, 1968). The clinical appearance of the tumour was similar to that of the 2 other tumours described here, and the histology report identical.

### DISCUSSION

It is the custom among several tribes (Makua, Makonde, Mawia, Yao) in the south of Tanzania to pierce the centre of the upper lip of the womenfolk at puberty and to introduce successively larger plugs of wood which are worn throughout adult life.

In all the 3 cases recorded here, squamous carcinoma arose in an annular fashion around the margin of the distended orifice in the upper lip. Details of these cases with special reference to their surgical management have been reported more fully elsewhere (Cubey, 1974).

* Present address: Department of Surgery, Leeds University, Leeds LS2 9JT.

FIG. 1.—A large and typical lip plug.

The implications of lip plug carcinoma, to which no other reference has been found, are discussed here with respect to its possible aetiology in the context of the pattern of tumour incidence in the geographical area in which it occurs.

(*a*) *Chemical carcinogenesis*

It seems reasonable to suppose that a process involving chemical carcinogenesis is operative in the pathogenesis of lip plug carcinoma, though the likelihood of a specific carcinogen common to all the cases here described is diminished by the observation that the plug used is cut from the wood of different trees, ebony heartwood in the case of the Makua tribe and sapwood from a different species of tree in the case of the Makonde tribe.

(*b*) *Trauma*

The possible role of previous trauma in carcinogenesis generally is exemplified by the well documented carcinoma of the

FIG. 2.—Case 1. Lip plug carcinoma.

skin arising in a scar from a previous injury or burn. The commonest of all malignancies in southern Tanzania is the carcinoma arising from the skin of the lower leg at the margin of a chronic non-healing " tropical " ulcer, in the pathogenesis of which trauma plays a possible initial part, though malnutrition and chronic local sepsis are major factors in its maintenance. However, the presence of some specific carcinogen in the discharge from this type of ulcer may possibly be suggested by the comparative rarity of malignant change in the scarcely less common chronic gravitational or " varicose " ulcer encountered in Western countries at the same anatomical site.

(c) *Presence of foreign body; chronic ulceration*

In malignant tropical ulcer, the persistent attempt of the epidermis to heal the ulcer by hyperplasia, which may be

FIG. 3.—Case 2. Lip plug carcinoma.

successful on several occasions only to be followed by further breaking down of the ulcer, eventually gives way to a neoplastic change. Similarly, in lip plug carcinoma, the persistent presence of the lip plug in itself causes ulceration and also frustrates the prolonged attempts by the epidermis and buccal epithelium to heal the ulcer by cellular hyperplasia, which eventually therefore gives way to a neoplastic change.

On the other hand, a similar state of affairs in a neighbouring area of skin, that of the ear lobe which is also pierced in young women of these tribes and often kept patent by stalks of dried grass, has not been observed to give rise to neoplasia, but frequently on the other hand to abundant keloid formations up to 3 or 4 cm in diameter, which hang as spherical masses from the ear lobe and are prone to recur after excision but show no ulceration or characteristics of malignancy. Keloid has not been observed in relation to lip plugs.

FIG. 4.—Case 2. After excision of lip plug carcinoma and repair by Abbe flap.

Malignant tropical ulcer, together with carcinoma of the bladder associated with *Schistosoma haematobium* infestation, and with primary hepatoma, forms a triad of the 3 commonest malignant tumours seen in this area, as shown by the monthly cancer returns personally notified to the M.R.C. over a 5-year period 1966–71. All 3 may be related to chronic overgrowth of cells in response to a local noxious agent (and lip plug carcinoma appears to be another such example in this area), and are together far commoner than all others occurring in southern Tanzania, and do not occur in this form in the Western world. Even so, the overall incidence of cancer here, as in the developing countries of East Africa generally, is considerably lower than in the Western world, causing for example less than 4% of deaths in Uganda (Templeton, 1973). Out of 7347 malignant tumours, Templeton recorded 37 malignant tumours of the lips. On this basis, carcinoma of the lip is less common in Uganda than squamous carcinoma affecting preferentially the lower lip in males in Western Europe, where although there are much lower levels of actinic radiation there is on average much less protective melanin pigmentation in the skin of the lip. Templeton's 37 cases included a few cases of Burkitt lymphoma and Kaposi sarcoma but no reference was made to lip plug carcinoma.

One clear-cut example reported from a Western country of squamous carcinoma arising in relation to a persistent foreign body is that caused by the neglected vaginal pessary (Russell, 1961). Russell found that in 6 out of 8 cases of primary cancer of the vagina the patient had worn a ring pessary for many years and the distribution of the cancer suggested that chronic irritation from the pessary was an important aetiological factor. He also reported ulcerating vaginitis without neoplasia in a further 5 such patients. This appears to be a close parallel to lip plug carcinoma, the likely aetiology of which may be summarized:

Original injury
↓
Scar + Foreign body
↓
Chronic ulceration (prolonged cellular hyperplasia) + ?specific carcinogen
↓
Neoplasia

REFERENCES

CUBEY, R. BEVIS (1974) Lip-plug Carcinoma and its Management by Modified Abbe Flap. *Br. J. plast. Surg.* In the press.
RUSSELL, J. K. (1961) The Dangerous Vaginal Pessary. *Br. med. J.*, ii, 1595.
TEMPLETON, A. C. (1973) *Tumours in a Tropical Country.* (*Recent Results in Cancer Research.*) Berlin: Springer-Verlag.